# Sex differences in the expression of the endocannabinoid system within V1M cortex and PAG of Sprague Dawley rats

**DOI:** 10.1186/s13293-021-00402-2

**Published:** 2021-11-08

**Authors:** Aidan Levine, Erika Liktor-Busa, Austin A. Lipinski, Sarah Couture, Shreya Balasubramanian, Sue A. Aicher, Paul R. Langlais, Todd W. Vanderah, Tally M. Largent-Milnes

**Affiliations:** 1 Department of Pharmacology, University of Arizona, 1501 N. Campbell Ave., Life Sciences North Rm 621, Tucson, AZ 85724 USA; 2 Endocrinology Division, Department of Medicine, University of Arizona, Tucson, AZ 85724 USA; 3 Department of Chemical Physiology & Biochemistry, Oregon Health & Science University, Portland, OR 97239 USA

**Keywords:** Sex differences, Endocannabinoids, 2-Arachidonoylglycerol, Anandamide, Migraine, Pain, Proteomics, Periaqueductal grey

## Abstract

**Background:**

Several chronic pain disorders, such as migraine and fibromyalgia, have an increased prevalence in the female population. The underlying mechanisms of this sex-biased prevalence have yet to be thoroughly documented, but could be related to endogenous differences in neuromodulators in pain networks, including the endocannabinoid system. The cellular endocannabinoid system comprises the endogenous lipid signals 2-AG (2-arachidonoylglycerol) and AEA (anandamide); the enzymes that synthesize and degrade them; and the cannabinoid receptors. The relative prevalence of different components of the endocannabinoid system in specific brain regions may alter responses to endogenous and exogenous ligands.

**Methods:**

Brain tissue from naïve male and estrous staged female Sprague Dawley rats was harvested from V1M cortex, periaqueductal gray, trigeminal nerve, and trigeminal nucleus caudalis. Tissue was analyzed for relative levels of endocannabinoid enzymes, ligands, and receptors via mass spectrometry, unlabeled quantitative proteomic analysis, and immunohistochemistry.

**Results:**

Mass spectrometry revealed significant differences in 2-AG and AEA concentrations between males and females, as well as between female estrous cycle stages. Specifically, 2-AG concentration was lower within female PAG as compared to male PAG (*p = 0.0077); female 2-AG concentration within the PAG did not demonstrate estrous stage dependence. Immunohistochemistry followed by proteomics confirmed the prevalence of 2-AG-endocannabinoid system enzymes in the female PAG.

**Conclusions:**

Our results suggest that sex differences exist in the endocannabinoid system in two CNS regions relevant to cortical spreading depression (V1M cortex) and descending modulatory networks in pain/anxiety (PAG). These basal differences in endogenous endocannabinoid mechanisms may facilitate the development of chronic pain conditions and may also underlie sex differences in response to therapeutic intervention.

**Supplementary Information:**

The online version contains supplementary material available at 10.1186/s13293-021-00402-2.

## Background

Chronic pain disorders affect roughly 20% of adults in the United States, with an increased prevalence in the female population [1]. While the pathogenesis and treatment for certain chronic pain states (i.e., rheumatoid arthritis), have been well documented [2], those of functional chronic pain disorders, such as migraine and fibromyalgia remain elusive. One proposed hypothesis for the pathogenesis of functional pain disorders is the theory of Clinical Endocannabinoid Deficiency (CED) [3].

CED describes chronically low levels of the two main endogenous cannabinoids (eCBs), 2-arachidonoylglycerol (2-AG) and anandamide (AEA), in platelets and the CSF of migraine patients. The endocannabinoid system (ECS) includes the enzymes responsible for the synthesis and degradation of 2-AG and AEA which are primarily synthesized from cellular lipid membranes via the enzymes diacylglycerol lipase (DAGL) and *N*-acyl-phosphatidylethanolamine hydrolyzing phospholipase D (NAPE-PLD), respectively [4, 5]. The degradation of 2-AG is primarily accomplished via monoacylglycerol lipase (MAGL) and α/β-hydrolase-6 (ABHD6), and that of AEA is performed primarily by fatty acid amide hydrolase (FAAH) [4]. Expression of the ECS components is brain region and cell-type selective. For example, expression of MAGL is reported to be mainly presynaptic at extrasynaptic regions rich in CB_1_R [6] whereas DAGL and ABHD6 are primarily post-synaptic [7, 8]. DAGL, MAGL, and ABHD6 enzymes are reportedly expressed by glial cells [9]. Both 2AG and AEA act on the cannabinoid receptors CB1 and CB2. CB1 represents the primary cannabinoid receptor in the central nervous system (CNS) [4], while CB2 receptors are reported in glial cells and in some neurons [10]. Activation of these receptors reduces neurotransmitter release at glutamatergic and GABA-ergic synapses and inhibits inflammatory responses [11–15].

While the mechanisms driving functional pain disorders remain debated, there is general agreement within the literature of the importance of pain-relevant regions in the central nervous system [16–18]. Regions of interest in pain sensation and modulation include several cortical areas as well as the periaqueductal grey (PAG). Canonically, pain signals are interpreted and modulated by cortical regions [19, 20], which then project to the PAG. Neurons in the PAG project to the rostral ventral medulla (RVM), which in turn sends efferents to the dorsal horn to modulate afferent pain sensations from the spinal cord and trigeminal nucleus caudalis (Vc), which receives nociceptive signals via the trigeminal nerve (TG) [21–23]. During migraine with aura, the cortical spreading depression events originating in the V1M cortex are thought to underlie visual aura; these CSD events also lead to indirect activation of the PAG [18]. Thus, the PAG and V1M cortex represent relevant areas of interest for examination of role of the ECS in pain signaling.

As functional pain states, including migraine, show an increased prevalence in the female population [1, 24, 25], physiologic differences in expression and localization of ECS components between males and females may underscore the female prevalence of these disorders. Thus, these studies tested whether ECS expression differed between male and female rats in a regionally selective manner in the descending pain pathways that are implicated in chronic pain disorders. Utilizing mass spectrometry, the levels of 2-AG and AEA were quantified for male and female rats in the V1M Cortex, PAG, Vc, and TG. Several reports have indicated that both 2-AG and AEA levels may fluctuate according to female estrous cycle stage [26, 27]. Therefore, an additional analysis of 2-AG and AEA levels within the CNS of intact females was performed according to the stages of the estrous cycle. The ratio of 2-AG:AEA was analyzed as well between sex and across estrous cycle stages, as fluctuations in the absolute concentration of the individual eCBs may not reflect their combined physiologic effects. DAGL, MAGL, ABHD6, NAPE-PLD, FAAH, CB1, and CB2 expression and localization were determined using immunohistochemistry of the PAG, a nucleus implicated in the descending pain modulatory pathways, using the occipital cortex (V1M), a site often associated with the induction of cortical spreading depression events linked to migraine aura, for comparison. Follow-up confirmation of PAG immunohistochemical findings was performed via unbiased proteomics. Levels of the eCBs, as well as enzyme and receptor expression and localization, varied by central nervous system region and sex. These data suggest differences between the male and female ECS in brain regions associated with pain modulation that may underlie sex differences in some pain etiologies, as well as pharmacological responsivity.

## Materials and methods

### Animals

Female and male Sprague Dawley rats (7–8 weeks old) were purchased from Envigo (Indianapolis, IN) and housed in a climate-controlled room on a regular 12/12 h light/dark cycle with lights on at 7:00 am with food and water available ad libitum. Normal feeding and weight gain was observed for all animals. Animals were housed three to a cage. Males and females were housed on separate racks within the same room. As stressful conditions have been associated with alterations to the ECS [28], animals were handled daily for a minimum of 5 min each and housed within the vivarium for at least one week prior to harvest. Daily vaginal smear and microscopic evaluation for estrous stage was performed on females as previously described [29]; researchers scored vaginal smears with greater than 50% nucleated epithelial cells as proestrus, greater than 50% cornified epithelial cells as estrus, and greater than 50% leukocytes as diestrus. Metestrus was identified as having between 30 and 40% of each of the above cell types. Animals were ensured to have progressed normally through the estrous stages (proestrus, estrus, metestrus, diestrus) for at least 8 days prior to tissue harvest and vaginal cytology was assessed at approximately 0930 h each day, with tissue harvest taking place at 1100 h. All procedures were performed during the 12-h light cycle and according to the policies and recommendations of the International Association for the Study of Pain, the NIH guidelines for laboratory animals, with IACUC approval from the University of Arizona. Animal numbers required to achieve statistical power for each assay were determined by G.Power3.1 in alignment with NIH policy (NOT-OD-15-102) such that differences of 20% were detected with 80% power at a significance level of 0.05 [30].

### Harvest of tissue samples

Each rat was anesthetized with ketamine:xylazine mix (80:10 mg/kg, i.p.), then transcardially perfused with ice-cold 0.1 M phosphate buffer at a rate of 3.1 ml/min for 10 min. After decapitation, spatially discrete brain regions involved in pain signaling [occipital V1M cortex (Ct), ventrolateral periaqueductal gray (vlPAG), trigeminal nucleus caudalis (Vc), and trigeminal ganglia (TG)] were harvested, flash frozen in liquid nitrogen, and stored at − 80 °C until preparation.

### Immunohistochemistry and microscopy

Rats were anesthetized and transcardially perfused as above, followed by transcardial perfusion with 4% paraformaldehyde (PFA) diluted in 0.1 M phosphate buffer. Brains were removed and post-fixed 2 h in 4% PFA, then transferred to 30% sucrose in PBS overnight. Brains were then flash frozen and cut in 40-micron coronal slices using a microtome (Microm HM 525). Free-floating sections were then washed in 0.1 M Tris saline solution (TS) before and after every incubation. First, sections were incubated for 1 h with 0.5% bovine serum albumin (BSA) in 0.1 M TS as a blocking solution, then adjacent sections through each brain region of interest were incubated for 48 h in primary antibody dilutions as follows: chicken anti-glial fibrillary acidic protein (1:500, Invitrogen PA1-10004), mouse anti-Iba1 (1:300, Invitrogen MA5-27726), goat anti-MAGL (1:250, Abcam 77398), goat anti-DAGL (1:500, Abcam 81984), rabbit anti-CB1R (1:250, Abcam 23703), rabbit anti-FAAH (1:135, Invitrogen PA5-52433), rabbit anti-NAPE-PLD (1:500, Invitrogen PA5-72923), or rabbit anti-ABHD6 (1:250, Invitrogen PA5-38999). Primary antibody dilutions were performed in 0.1% BSA/0.25% Triton in 0.1 M TS. Tissue sections were rinsed thoroughly in TS then incubated in the dark for 2 h with the appropriate fluorescently conjugated secondary antibodies as follows (all secondary dilutions were 1:400): AlexaFluor™ 488 Goat anti-chicken (Invitrogen A-11039), AlexaFluor™ 647 Goat anti-chicken (Invitrogen A-21449), AlexaFluor™ 568 Donkey anti-mouse (Invitrogen A-10037), AlexaFluor™ 488 Donkey anti-rabbit (Invitrogen A-21206), or AlexaFluor™ 647 Donkey anti-goat (Invitrogen A-32849). Secondary antibody dilutions were performed in 0.1% BSA in 0.1 M TS. Tissue sections were then washed with 0.1 M TS followed by 0.1 M PB. Finally, tissue sections were incubated for 30 min in NeuroTrace™ 435/455 Blue Fluorescent Nissl Stain (1:200, Invitrogen N-21479) in 1× PBS. Slices were then mounted and coverslipped with ProLong™ Gold antifade reagent w/o DAPI. Epifluorescence images were obtained on an ECHO Revolve microscope and high-resolution images were obtained on a Zeiss Elyra S.1 structured illumination microscope. Image analysis was performed with either ZEN 3.1 Blue software (Carl Zeiss. Jena, Germany) or FIJI ImageJ (https://imagej.net/Fiji) [31]. Within Zen Blue, z-stacks were first processed with a structured illumination Fourier transform followed by a channel alignment algorithm. Z-stacks were then resolved into high-resolution 2D images via extended depth of focus (EDF) and denoising processing. The colocalization function within the software was then utilized to quantify fluorescent pixels, and thresholds were set to include appropriate pixel intensities. Colocalization and pixel area data were then extracted from these images. Within ImageJ, semi-quantitative analysis of protein expression was performed as previously described [32]. As the proteins of interest exist within extracellular and perisynaptic spaces, whole slide images were analyzed. To accommodate for differences in staining, fluorescence intensity, and neuronal soma captured between users, a Nissl stain for soma was used as a standard to correct image intensity as previously described [33]. Fixed regions of tissue were imaged and quantified by an observer blind to experimental condition; threshold was similar for all samples and results are expressed as relative immunoreactivity corrected to mean Nissl immunoreactivity. All image analyses were performed in triplicate.

### Quantification of 2-AG and AEA by LC–MS

The brain samples for LC–MS were purified by organic solvent extraction, as described by Wilkerson et al. [34]. Briefly, tissues were harvested, snap-frozen in pre-weighted tubes, and stored at − 80 °C. On the day of processing, tissues were weighed and homogenized in 1 ml of chloroform/methanol (2:1 v/v) supplemented with phenylmethylsulfonyl fluoride (PMSF) at 1 mM final concentration to inhibit the degradation by endogenous enzymes. Dounce homogenizer was used for homogenization. Homogenates were then mixed with 0.3 ml of 0.7% w/v NaCl, vortexed, and then centrifuged for 10 min at 3200×*g* at 4 °C. The aqueous phase plus debris was collected and extracted two more times with 0.8 ml of chloroform. The organic phases from the three extractions were pooled and internal standard was added to each sample. Mixed internal standard solutions were prepared by serial dilution of AEA-d4 and 2-AG-d5 in acetonitrile. The organic solvents were evaporated under nitrogen gas and 6 μl of 30% glycerol in methanol per sample was added before evaporation. Dried samples were reconstituted with 0.2 ml of chloroform and mixed with 1 ml of ice-cold acetone to precipitate proteins. The mixtures were then centrifuged for 5 min at 1800×*g* at 4 °C. The organic layer of each sample was collected and evaporated under nitrogen.

Analysis of 2-AG and AEA was performed on an Ultivo triple quadrupole mass spectrometer combined with a 1290 Infinity II UPLC system (Agilent, Palo Alto, CA). The instrument was operated in electrospray positive mode with a gas temperature of 150 °C at a flow of 5 l/min, nebulizer at 15 psi, capillary voltage of 4500 V, sheath gas at 400 °C with a flow of 12 l/min and nozzle voltage of 300 V. Transitions monitored were 348.3→287.3 and 62, 352.3→287.4 and 65.9, 379.3→287.2 and 269.2, and 384.3→287.2 and 296.1 for AEA, AEA-d4, 2-AG, and 2-AG-d5. The first fragment listed was used for quantification and the second fragment was used for confirmation. The first 3 min of analysis time was diverted to waste. Chromatographic separation was achieved using an isocratic system of 21% 1 mM ammonium fluoride and 79% methanol on an Acquity UPLC BEH C-18 1.7 μm, 2.1 × 100 mm column (Waters, Milford, MA) maintained at 60 °C. After each injection, the column was washed with 90% methanol for 1 min then re-equilibrated for 5 min prior to the next injection. Samples were maintained at 4 °C. Mixed calibration solutions were prepared by serial dilution of AEA and 2-AG stock solutions in 80% acetonitrile. Calibration curves were prepared for each analysis by adding 10 µl internal standard solution to 20 µl standard solution. Prior to sample analysis, 200 µl of 80:20 acetonitrile:water was added to dried samples which were then vortexed and sonicated. The samples were centrifuged at 15,800×*g* at 4 °C for 5 min. Supernatant was transferred to autosampler vials and 5 µl was injected for analysis.

### Proteome analysis of naive PAG

To determine sex differences in the PAG proteome of rats, tissue samples were harvested as described above. The samples were lysed in RIPA-style lysis buffer, containing phosphatase and protease inhibitors (BiMake). The samples were sonicated three times, with 1-s pulses, followed by centrifugation at 15,000×*g* for 10 min at 4 °C. The supernatant was collected, BCA assay (Pierce™ BCA Protein Assay Kit, Thermo Scientific) was performed to determine the protein content, then 200 μg of the lysate supernatant was separated on a 10% SDS-PAGE gel and stained with Bio-Safe Coomassie G-218250 Stain. Each lane of the SDS-PAGE gel was cut into six slices. The gel slices were subjected to trypsin digestion and the resulting peptides were purified by C18-based desalting exactly as previously described [35, 36].

HPLC–ESI–MS/MS was performed in positive ion mode on a Thermo Scientific Orbitrap Fusion Lumos tribrid mass spectrometer fitted with an EASY-Spray Source (Thermo Scientific, San Jose, CA). NanoLC was performed exactly as previously described [35, 36]. Tandem mass spectra were extracted from Xcalibur ‘RAW’ files and charge states were assigned using the ProteoWizard 3.0 msConvert script using the default parameters. The fragment mass spectra were searched against the *Mus musculus* and *Rattus norvegicus* SwissProt_2018_01 databases using Mascot (Matrix Science, London, UK; version 2.6.0) using the default probability cut-off score. The search variables that were used were: 10 ppm mass tolerance for precursor ion masses and 0.5 Da for product ion masses; digestion with trypsin; a maximum of two missed tryptic cleavages; variable modifications of oxidation of methionine and phosphorylation of serine, threonine, and tyrosine. Cross-correlation of Mascot search results with X! Tandem was done with Scaffold (version Scaffold_4.8.7; Proteome Software, Portland, OR, USA). Probability assessment of peptide assignments and protein identifications were made using Scaffold. Only peptides with ≥ 95% probability were considered.

Progenesis QI for proteomics software (version 2.4, Nonlinear Dynamics Ltd., Newcastle upon Tyne, UK) was used to perform ion-intensity based label-free quantification. In brief, in an automated format, .raw files were imported and converted into two-dimensional maps (*y*-axis = time, *x*-axis = *m/z*) followed by selection of a reference run for alignment purposes. An aggregate data set containing all peak information from all samples was created from the aligned runs, which was then further narrowed down by selecting only + 2, + 3, and + 4 charged ions for further analysis. The samples were then grouped and a peak list of fragment ion spectra from only the top eight most intense precursors of a feature was exported in Mascot generic file (.mgf) format and searched against the *Mus musculus* and *Rattus norvegicus* SwissProt_2018_01 database using Mascot (Matrix Science, London, UK; version 2.4). The search variables that were used were: 10 ppm mass tolerance for precursor ion masses and 0.5 Da for product ion masses; digestion with trypsin; a maximum of two missed tryptic cleavages; variable modifications of oxidation of methionine and phosphorylation of serine, threonine, and tyrosine; 13C = 1. The resulting Mascot .xml file was then imported into Progenesis, allowing for peptide/protein assignment, while peptides with a Mascot Ion Score of < 25 were not considered for further analysis. Protein quantification was performed using only non-conflicting peptides and precursor ion-abundance values were normalized in a run to those in a reference run (not necessarily the same as the alignment reference run). Principal component analysis and unbiased hierarchal clustering analysis (heat map) was performed in Perseus [37, 38] while Volcano plots were generated in RStudio.

### Data analysis and statistics

GraphPad Prism 9.0 software (GraphPad Software) was used for statistical analysis. Unless otherwise stated, the data were expressed as mean ± SEM. Mass spectrometry data were compared between sexes via Mann–Whitney rank-sum test with correction for multiple comparisons via the Bonferroni–Dunn method. Mass spectrometry data compared between estrous cycle stages within female animals were analyzed via two-way ANOVA with Tukey’s post hoc analysis. Immunohistochemical data were compared between sex and region via two-way ANOVA with Sidak’s post hoc analysis. Differences were considered significant if *p* ≤ 0.05 to give 80% power (GPower 3.1). Statistics were performed according to recommendations of the UA Stats Consulting laboratory.

## Results

### LC–MS analysis of endocannabinoids (eCBs) in female and male tissue

**Fig. 1:**
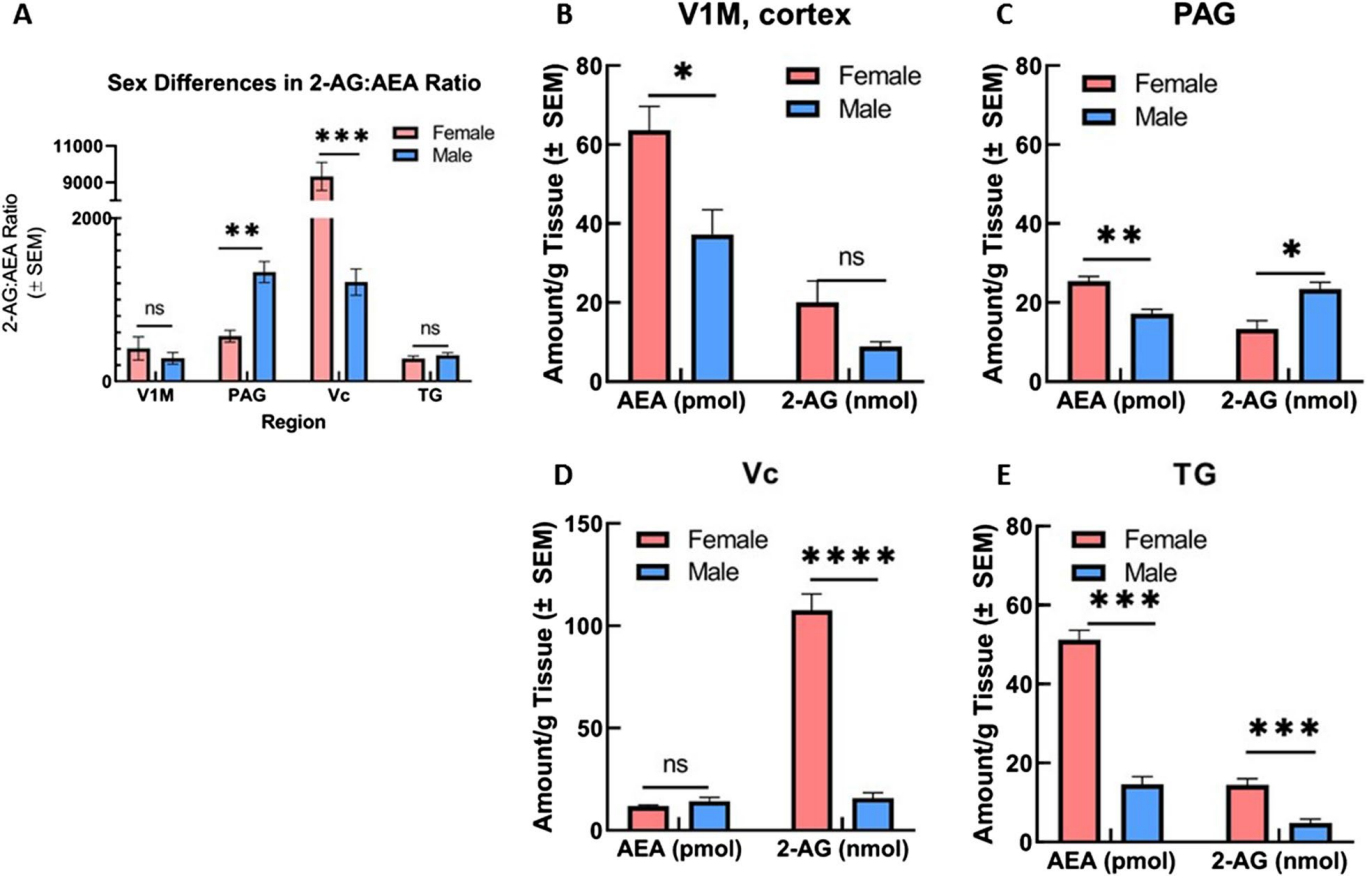
Regional and sex-differences in the level of two main endocannabinoids, 2AG and AEA. Regional and sex-differences of 2-AG and AEA levels were observed in CNS areas (cortex, PAG, Vc, and TG) harvested from naïve female and male SD rats. **A** Comparison of ratio of 2-AG:AEA between sexes and across CNS regions. Within PAG, ratio of 2-AG:AEA was significantly increased in males as compared to females (**p = 0.0049). Female ratio of 2-AG:AEA within Vc was significantly greater than the ratio observed in males (***p < 0.0001). No significant differences observed within V1M cortex or TG. **B** Comparison of AEA and 2-AG levels between sexes within V1M cortex. Female AEA concentration observed as significantly greater than male AEA concentration (*p = 0.035). No significant sex difference observed for 2-AG concentration. **C** Comparison of AEA and 2-AG levels between sexes within periaqueductal grey (PAG). Female AEA concentration within PAG was significantly greater than male AEA concentration (**p = 0.0035), Female 2-AG concentration observed to be significantly decreased as compared to male 2-AG concentration within PAG (*p = 0.015). **D** Comparison of AEA and 2-AG levels between sexes within Vc. Female 2-AG concentration was detected at significantly higher levels than male 2-AG concentration (****p = 0.0008). No significant differences observed for AEA concentration between sexes. **E** Comparison of AEA and 2-AG levels between sexes within trigeminal nerve (TG). Female AEA concentration was significantly greater than male AEA concentration (***p = 0.0056), Female 2-AG concentration also observed as significantly greater than male 2-AG concentration (***p = 0.004). AEA concentrations reported as picomole/gram tissue, 2-AG concentrations reported as nanomole/gram tissue. *n* = 5 for all male regions, *n* = 20 females (5/estrous stage) for all female regions

We examined the total level of the primary eCBs, AEA and 2-AG, in V1M cortex, PAG, Vc, and TG of male (*n* = 5) and female (*n* = 20, estrous stages pooled) rats using LC–MS. In both sexes, 2-AG was detected at higher concentrations than AEA (nmol/g tissue versus pmol/g tissue), in accordance with the findings of others [39] (Fig. 1). Comparison between the sexes was also performed for the ratio of 2-AG:AEA within CNS regions (Fig. 1A). Significant differences in AEA concentration between sexes were observed within V1M cortex, PAG, and TG (Fig. 1B–E). Female AEA concentration was detected at significantly greater levels than male AEA concentration within these regions, with the greatest difference observed within the TG region (*p* = 0.019, female vs male [AEA] V1M cortex; *p* = 0.0005, female vs male [AEA] PAG; *p* = 0.00001 female vs male [AEA] TG; Mann–Whitney rank-sum test).

Comparison of CNS 2-AG concentrations between male and female rats revealed significant differences within PAG, Vc, and TG regions (Fig. 1B–E). Female 2-AG concentration in Vc and TG regions was detected at significantly greater levels than male 2-AG concentration within Vc and TG (*p* < 0.00001, female vs male [2-AG] Vc; *p* = 0.002 female vs male [2-AG] TG; Mann–Whitney rank-sum test). Within PAG, however, female 2-AG concentration was observed to be significantly decreased as compared to male PAG 2-AG concentration (*p* = 0.0077, Mann–Whitney rank-sum test). As a post hoc analysis, the ratios of 2-AG:AEA between the sexes within these regions was quantified (Fig. 1A). Despite the observed sex differences for 2-AG and AEA levels within V1M cortex and TG, no significant difference was observed for 2-AG:AEA ratio between male and female rats within these regions. Within PAG, the ratio of 2-AG:AEA was significantly decreased for female rats as compared to males (*p* = 0.0049, Mann–Whitney rank-sum test). The ratio of 2-AG:AEA within Vc was observed to be significantly greater for females as compared to males (*p* < 0.0001, Mann–Whitney rank-sum *t*-test).

### LC–MS analysis of endocannabinoids (eCBs) in estrous staged female tissue

**Fig. 2:**
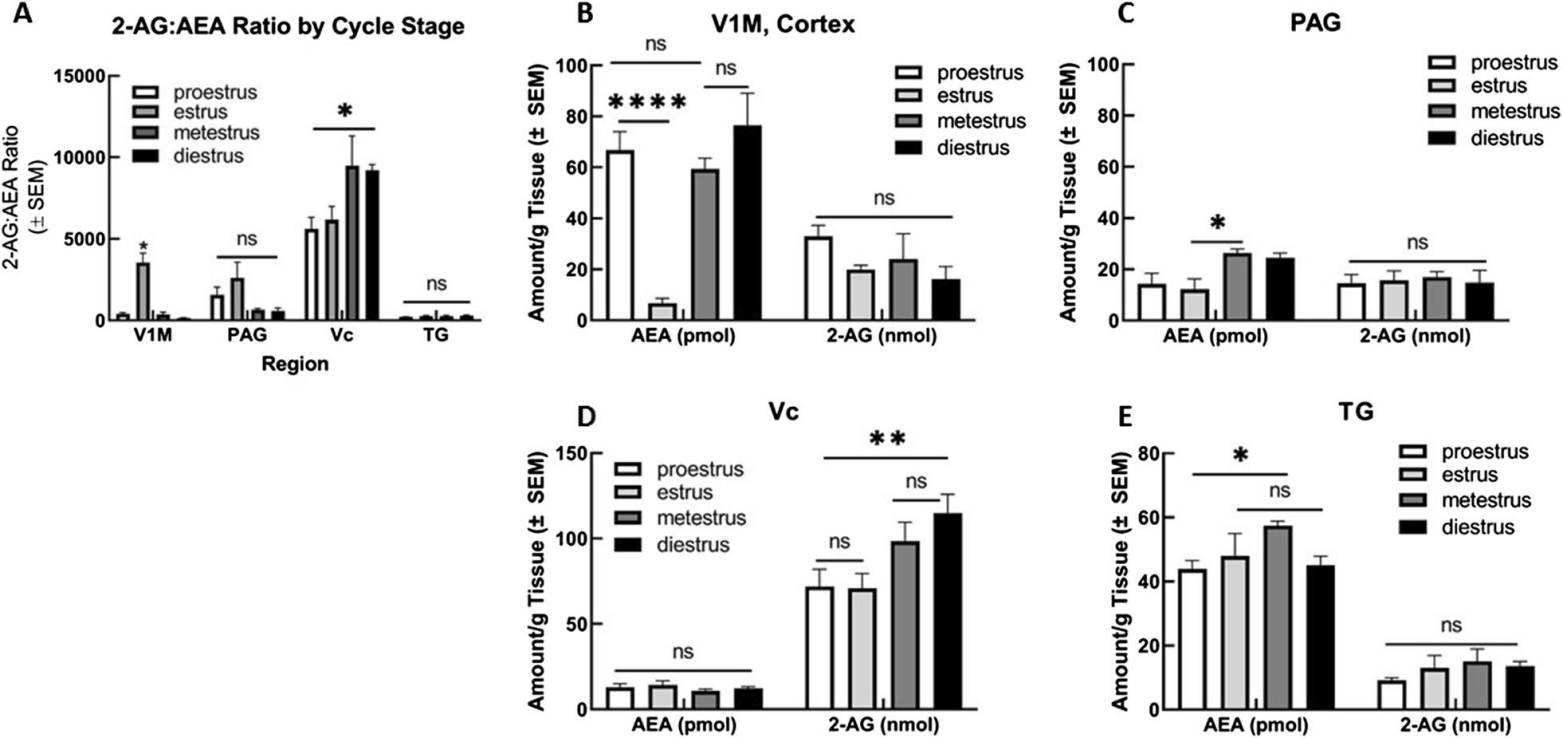
Estrous stage differences in 2-AG and AEA concentrations within female CNS regions. Differences in 2-AG and AEA concentrations were observed between female animals harvested according to varying estrous cycle stages (proestrus, estrus, metestrus, diestrus). **A** Comparison of ratio of 2-AG:AEA across CNS regions according to cycle stage. Within V1M cortex, females in estrus stage showed significantly greater 2-AG:AEA ratio as compared to proestrus, metestrus, and diestrus (*p = 0.01). No significant differences observed for 2-AG:AEA ratio across cycle stages within PAG. Within Vc, ratio of 2-AG:AEA was significantly greater for diestrous females as compared to proestrous females (*p = 0.015). No significant differences observed for 2-AG:AEA ratio across cycle stages for TG. **B** Comparison of AEA and 2-AG concentrations across cycle stages within V1M Cortex. Females harvested within estrus stage showed significantly decreased AEA concentration as compared to proestrus, metestrus, and diestrus (****p < 0.0001). No other significant differences observed between cycle stage for cortical AEA concentration or 2-AG concentration. **C** Comparison of AEA and 2-AG concentrations across cycle stages within PAG. AEA concentration for estrous females was significantly decreased as compared to metestrous females (*p = 0.02). No significant differences observed for 2-AG concentration between cycle stages. **D** Comparison of AEA and 2-AG concentrations across cycle stages within Vc. No significant differences across cycle stage observed for AEA concentration. Diestrus 2-AG concentration observed to be significantly greater than proestrus and estrus 2-AG concentrations (**p = 0.0012). **E** Comparison of AEA and 2-AG concentrations across cycle stages within TG. Metestrus AEA concentration observed to be significantly greater than proestrus AEA concentration (*p = 0.0465). No significant differences observed for 2-AG concentrations across cycle stage. AEA concentrations reported as picomole/gram tissue, 2-AG concentrations reported as nanomole/gram tissue. *n* = 20 females were monitored and harvested according to their estrous stage with *n* = 5 groups for proestrus, estrus, metestrus, and diestrus

Estrous stage was monitored in females (*n* = 20) as detailed above, and tissue was harvested from females in proestrus, estrus, metestrus, and diestrus (*n* = 5/stage). We then examined eCB concentration within V1M cortex, PAG, Vc, and TG to analyze estrous stage dependence of 2-AG and AEA levels, as well as 2-AG:AEA ratio (Fig. 2A). Analysis via two-way ANOVA revealed significant estrous stage variation in AEA levels within V1M cortex (*F*(3,32) = 11.87, *p* < 0.0001). Tukey’s post hoc analysis between estrous cycle stages revealed V1M cortical tissue (Fig. 2B) harvested within estrus showed significantly decreased AEA concentration as compared to proestrus, metestrus, and diestrus (*p* < 0.0001 all comparisons, Tukey). Within PAG (Fig. 2C), AEA concentration was significantly decreased for estrus versus metestrus (*p* = 0.02, Tukey), despite failing to reach significance in the variation analysis. Tissue harvested within TG (Fig. 2E) from proestrous females also showed significantly decreased AEA concentration as compared to metestrous TG tissue (*p* = 0.0465, Tukey).

2-AG concentration was observed to be estrous stage dependent only within the Vc region (*F*(3,29) = 4.170, *p* = 0.0143). Within Vc, diestrous 2-AG concentration was detected at significantly greater levels than proestrous and estrous 2-AG concentrations (*p* = 0.0012, Tukey). As mentioned above, the ratio of 2-AG:AEA was compared as well between regions and across estrous cycle stages (Fig. 2A). The 2-AG:AEA ratio within the V1M cortex was significantly greater for estrous females as compared to proestrous, metestrous, and diestrous females on post hoc analysis (*p* = 0.01, Tukey). Within Vc, ratio of 2-AG:AEA was significantly greater for diestrous females as compared to proestrous females (*p* = 0.015, Tukey). No significant differences were observed for 2-AG:AEA ratio across cycle stages for PAG or TG.

### Immunohistochemistry of endocannabinoid system (ECS) in female and male tissue

To examine whether enzymatic differences might underlie the observed sex differences in endocannabinoid tone, we utilized immunohistochemistry to confirm LC–MS findings in the lPAG and V1M cortex (within factor). The lPAG was imaged at high resolution as a locus of the PAG implicated in trigeminal nociception [21–23]. Specifically, these studies evaluated ECS enzyme and receptor immunoreactivity in male and female rats (between factor). Distributions of NAPE-PLD, FAAH, DAGL, MAGL, ABHD6, CB_1_R, or CB_2_R in neurons, glia, or microglia were evaluated based on colocalization with cell-type specific markers (Nissl for neuronal soma, GFAP for astrocytes and Iba1 for microglia) as a secondary analysis of organizational differences. All analyses were conducted in a region of interest within the V1M cortex and lPAG with statistically similar areas and staining for Nissl, GFAP, and Iba1 (Additional file 1: Fig. 1)

#### AEA ECS

**Fig. 3:**
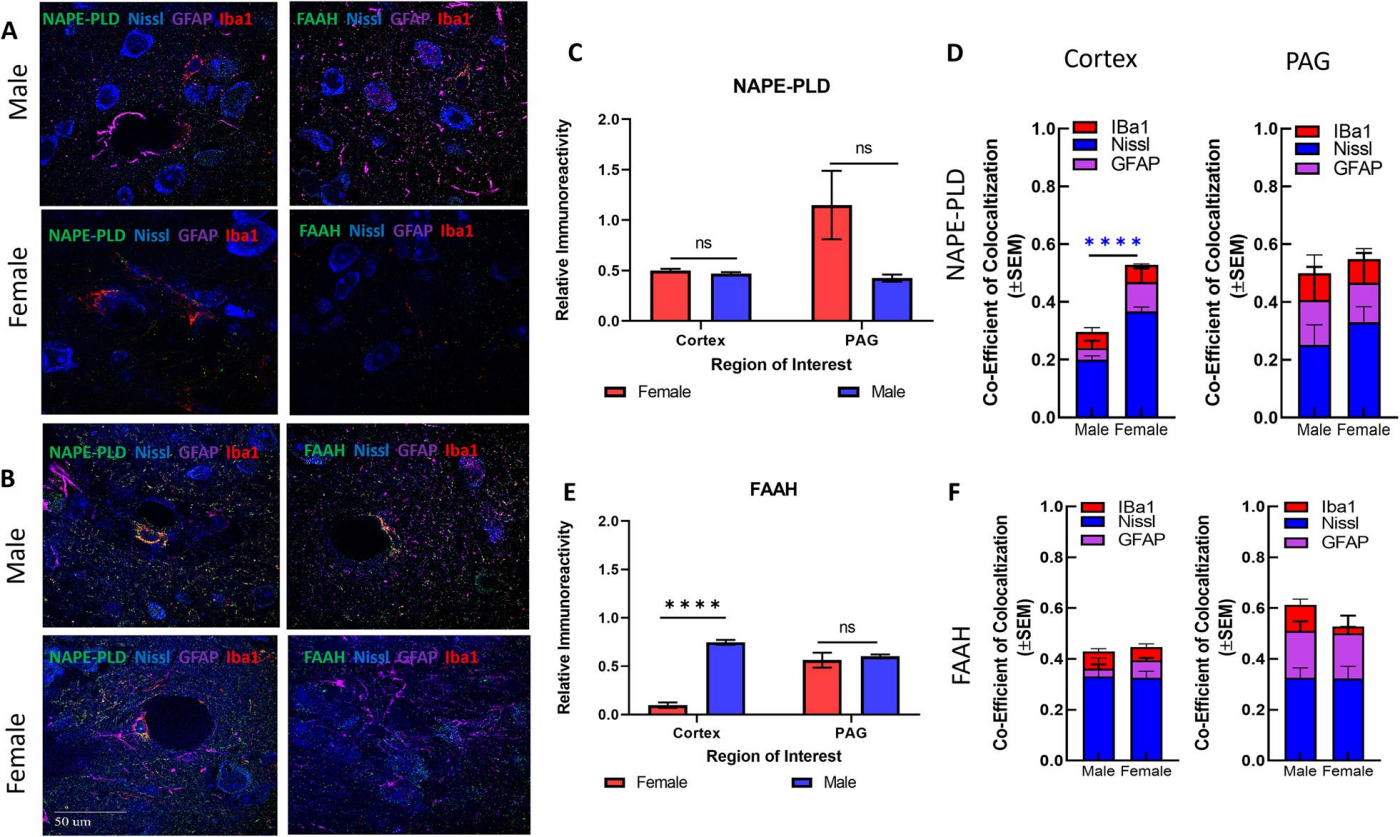
Immunohistochemical analysis of the AEA system. **A** Immunohistochemistry of naïve male and female cortex. Staining for NAPE-PLD, FAAH, Iba1, GFAP, and Nissl was performed. Original magnification ×63. **B** Immunohistochemistry of naïve male and female PAG. Staining for NAPE-PLD, FAAH, Iba1, GFAP, and Nissl was performed. Original magnification ×63. Scale on lower left image applies to all frames. **C** Measurement of relative area of IHC field occupied by NAPE-PLD staining pixels for each sex and region. No significant differences were observed between region or sex. **D** Measurement of co-efficient of colocalization for NAPE-PLD with IBa1, Nissl, and GFAP. NAPE-PLD colocalized with Nissl at significantly higher rates in female cortex vs male cortex (****p = 0.004). No significant differences observed in PAG. **E** Measurement of relative area of IHC field occupied by FAAH staining pixels for each sex and region. FAAH immunoreactivity was significantly higher in male cortex vs female cortex (****p = 0.003). **F** Measurement of co-efficient of colocalization for FAAH with IBa1, Nissl, and GFAP. No significant differences observed between region or sex. All calculations for co-efficient of colocalization were performed with respect to the relevant enzyme. *n* = 3/sex for each analysis

NAPE-PLD hydrolyzes NAPE to generate AEA, while degradation of AEA is controlled by FAAH-mediated hydrolysis. Immunohistochemistry was used to test the hypothesis that sex differences in cortical levels of AEA result from variability in the expression and localization of these enzymes (Fig. 3A, B). The interaction between region × sex was statistically significant for FAAH immunoreactivity (*F*(1,4) = 41.72, *p* = 0.003) with the post hoc analysis revealing a sex difference in the immunoreactivity within the V1M cortex such that male V1M FAAH was detected at higher levels than female (Sidak *p* < 0.00001, Fig. 3E). No significant differences were observed for the interaction between region × sex in analysis of NAPE-PLD immunoreactivity (Fig. 3C).

To determine whether NAPE-PLD was predominantly in neurons, astrocytes, or microglia, NAPE-PLD colocalization with Nissl, GFAP, and IBa1 was determined in both sexes for each CNS region (i.e., of all NAPE-PLD, how much colocalized with each marker; Fig. 3D). NAPE-PLD-ir colocalized with Nissl in males significantly more than in females within the V1M cortex (*F*(1, 4) = 144.0, *p* = 0.001; Sidak post hoc, *p* = 0.004). No sex differences in colocalization of NAPE-PLD with GFAP or Iba1 in the V1M cortex were observed. Comparison of NAPE-PLD colocalization within the lPAG revealed no significant sex differences between the cell-types (sex: *F*(1,4) = 0.12, *p* = 0.74, Fig. 3D). FAAH-ir colocalization with Nissl, GFAP, and Iba1 was not significantly different between sexes within either region (Fig. 3F).

Finally, in lPAG and V1M cortex, about 50% of NAPE-PLD immunoreactivity did not colocalize with Nissl, GFAP, or Iba1. Similar results were observed with FAAH immunoreactivity. This suggests that both NAPLD-PLD and FAAH are found in additional cellular regions, such as synaptic terminals.

#### 2-AG ECS

**Fig. 4:**
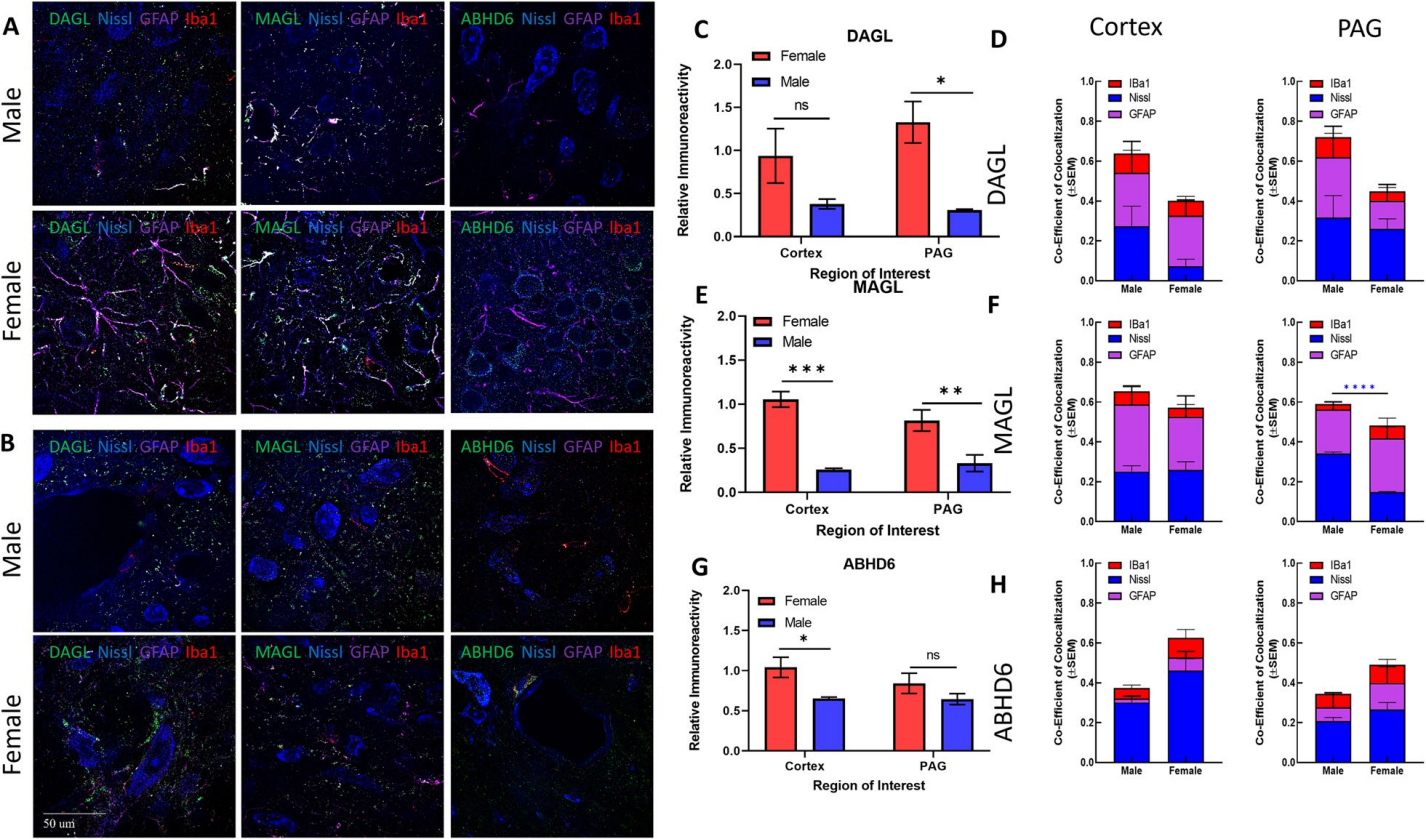
Immunohistochemical analysis of the AEA system. **A** Immunohistochemistry of naïve male and female V1M cortex. Staining for NAPE-PLD, FAAH, Iba1, GFAP, and Nissl was performed. Original magnification ×63. **B** Immunohistochemistry of naïve male and female PAG. Staining for NAPE-PLD, FAAH, Iba1, GFAP, and Nissl was performed. Original magnification ×63. **C** Measurement of relative area of IHC field occupied by NAPE-PLD staining pixels for each sex and region. Female NAPE-PLD immunoreactivity was significantly higher in PAG vs male PAG (*p = 0.02). **D** Measurement of co-efficient of colocalization for NAPE-PLD with IBa1, Nissl, and GFAP. NAPE-PLD colocalized with Nissl at significantly higher rates in female V1M cortex vs male V1M cortex (****p = 0.001). No significant differences observed in PAG. **E** Measurement of relative area of IHC field occupied by FAAH staining pixels for each sex and region. FAAH immunoreactivity was significantly higher in female PAG vs male PAG (***p = 0.0042) as well as female PAG vs female V1M cortex (^###^*p* = 0.002). **F** Measurement of co-efficient of colocalization for FAAH with IBa1, Nissl, and GFAP. No significant differences observed between region or sex. All calculations for co-efficient of colocalization were performed with respect to the relevant enzyme. *n* = 3/sex for each analysis

2-AG is synthesized from DAGs by DAGL and degraded by serine hydrolases, MAGL and ABHD6. Semi-quantitative immunohistochemistry of these three enzymes was performed to examine the hypothesis that 2-AG levels are lower in female PAG as compared to males resulting from differential enzyme expression (Fig. 4). DAGL was detected in both male and female samples with higher levels of DAGL found in the female lPAG versus V1M cortex. Similar patterns of overlap with Nissl, GFAP, and Iba1 were observed in both regions for female and male tissue. The interaction between region × sex was statistically significant for DAGL immunoreactivity (*F*(1,4) = 21.27, *p* = 0.0099) with the post hoc analysis revealing significantly greater immunoreactivity within the female lPAG (Sidak *p* = 0.0141, Fig. 4C).

MAGL immunoreactivity was detected in both male and female tissue within V1M cortex and lPAG (Fig. 4A, B). Statistical analysis revealed female tissue contained significantly greater levels of female MAGL immunoreactivity as compared to males for both regions (sex: *F*(1,4) = 195, *p* = 0.0002; female vs male cortex, Sidak *p* = 0.0004; female vs male lPAG, Sidak *p* = 0.0097). In the lPAG, males showed a significantly higher proportion of MAGL colocalization with Nissl as compared to females (*p* = 0.006, two-way ANOVA). No other significant differences were observed in MAGL colocalization.

ABHD6 expression was also detected in the tissue of both sexes (Fig. 4G). Female cortical tissue contained significantly greater levels of ABHD6 immunoreactivity as compared to males within the region (sex: *F*(1,4) = 9.703, *p* = 0.0357; female vs male cortex, Sidak *p* = 0.0425). No significant differences were observed in ABHD6 immunoreactivity within the lPAG. Analysis of co-immunoreactivity showed that ABHD6 overlapped primarily with Nissl in the lPAG; lPAG showed more colocalization of ABHD6 with GFAP than the V1M cortex in the female and male samples (Fig. 4H). In males, ABHD6 colocalized primarily with Nissl in lPAG.

Overall, the 2-AG-ECS analysis showed that females had higher levels of synthetic DAGL than males in the lPAG, as well as differential expression and localization the degradative enzyme MAGL. Notably, approximately 40–60% of DAGL, MAGL, and ABHD6 immunoreactivity did not colocalize with Nissl, GFAP, or Iba1. These data are suggestive of a sex-based difference in biological and circuit function for the 2-AG-ECS enzyme system within the PAG.

#### ECS receptors

**Fig. 5:**
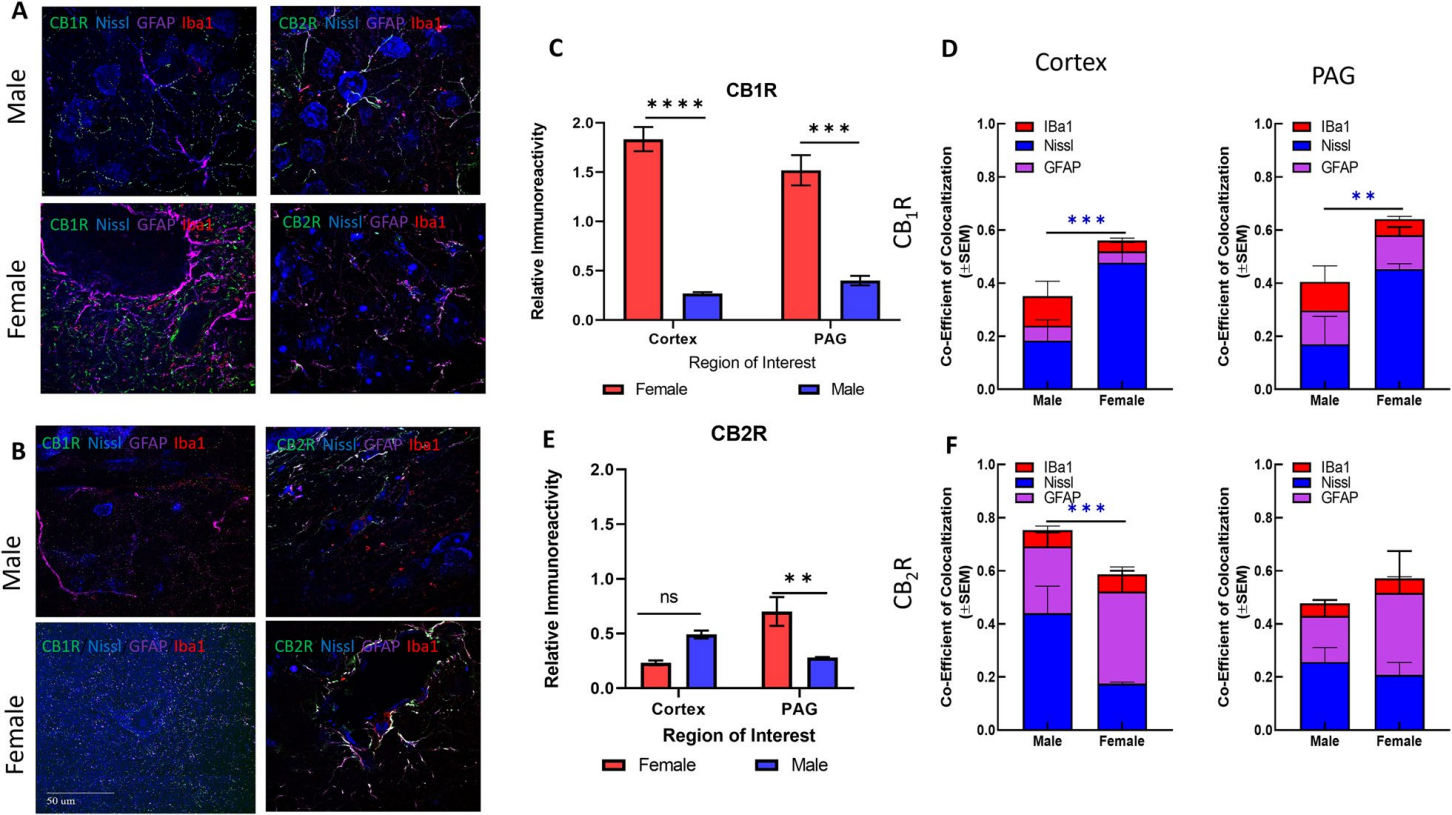
Immunohistochemical analysis of CB1R and CB2R. **A** Immunohistochemistry of naïve male and female cortex. Staining for CB1R, CB2R, Iba1, GFAP, and Nissl was performed. Original magnification ×63. **B** Immunohistochemistry of naïve male and female PAG. Staining for CB1R, CB2R, Iba1, GFAP, and Nissl was performed. Original magnification ×63. Scale on lower left image applies to all frames. **C** Relative immunoreactivity of CB1R staining revealed significantly greater staining for CB1R in female cortex and lPAG vs males for both regions (****p < 0.0001 female cortex vs male cortex, ***p = 0.001 female lPAG vs male lPAG). **D** Measurement of co-efficient of colocalization for CB1R with Iba1, Nissl, and GFAP. CB1R colocalization with Nissl was significantly higher in female cortex vs male cortex (***p = 0.008) and female PAG vs male PAG (**p = 0.01). **E** CB2R immunoreactivity was significantly higher in female lPAG vs male lPAG (**p = 0.0053). **F** Measurement of co-efficient of colocalization for CB2R with IBa1, Nissl, and GFAP. CB2R colocalization with Nissl in the male cortex was significantly greater than in female cortex (***p = 0.0178). All calculations for co-efficient of colocalization were performed with respect to the relevant enzyme. *n* = 3/sex for each analysis

AEA and 2-AG are partial and full agonists at the CBRs, respectively [39, 40], and changes in the levels of these endogenous ligands may be expected to vary with/or alter receptor density. Thus, CB_1_R and CB_2_R expression levels were compared between the sexes using immunohistochemistry (Fig. 5A, B). Analysis of CB_1_R immunoreactivity revealed significantly greater staining for CB_1_R for females versus males in both the V1M cortex and lPAG (sex: *F*(1,4) = 374.7, *p* < 0.0001; female vs male cortex, Sidak *p* < 0.0001; female vs male lPAG, Sidak *p* = 0.001). CB_2_R staining was similarly greater within the female lPAG as compared to the male lPAG. The interaction between region × sex was statistically significant (*F*(1,4) = 18.87, *p* = 0.0122), post hoc analysis revealed a sex difference in CB_2_R staining within the lPAG (Sidak *p* = 0.0053).

Analysis of colocalization revealed that female CB1R immunoreactivity had more overlap overall than male with Nissl, GFAP, or Iba1 independent of CNS region (Female total overlap: ~ 60%, male total overlap: ~ 40%, Fig. 5D). Within colocalization, CB_1_R showed statistically higher overlap with Nissl in female lPAG and V1M cortex when compared to males (*p* = 0.0178 female lPAG vs male lPAG; *p* = 0.008 female V1M cortex vs male V1M cortex; two-way ANOVA, Sidak); no differences were seen in the total area for which CB1R overlapped with either GFAP or Iba1 (Fig. 5D). Both males and females showed the highest percentage of overlap of CB_2_R with Nissl in the lPAG. No differences were observed in colocalization with GFAP or Iba1 immunoreactivity. Lastly, approximately 40–60% of CB1R and CB2R immunoreactivity did not colocalize with Nissl, GFAP, or Iba1, suggesting location of these receptors on other structures, such as presynaptic terminals (Fig. 5E, F).

### Proteomic analysis of female and male PAG samples

**Fig. 6:**
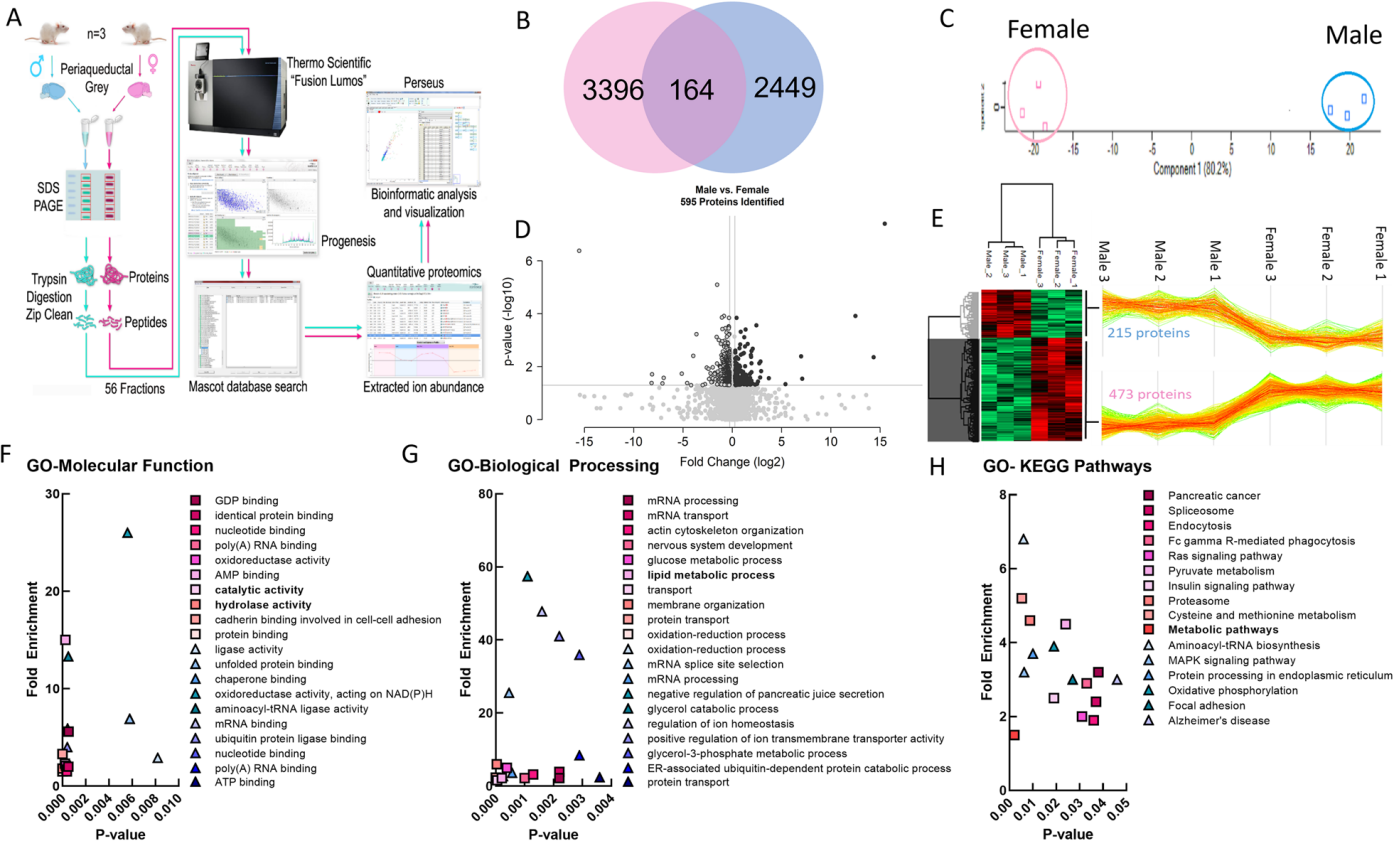
Proteomic analysis of naïve male and female PAG samples. **A** Schema of proteomic analysis. **B** Venn diagram of male vs. female sex differences in proteins detected. **C**–**E** Gene ontology (GO) enrichment analysis of the significantly affected proteins. **F** GO-molecular function analysis. **G** GO-biological processing analysis. **H** GO-KEGG pathway analysis

Seeking to further validate the sex differences observed on IHC and LC–MS, the global proteome between the male and female PAG was examined to identify sex-based patterns in this region relevant to descending pain modulation. The PAG was chosen as the primary focus as LC–MS and IHC studies had identified plausible sex differences in the ECS within this region. Quantitative proteomic analysis comparing global protein expression changes between naïve male and female PAGs identified 6009 total proteins across 72 fractions analyzed for the six biological samples (*n* = 3/sex). A total of 3396 proteins were more prevalent in female PAG with 2449 proteins showing prevalence in males (Fig. 6B, Additional file 2: Table 1). Of the 3396 proteins with female prevalence, 512 were present at statistically significantly higher levels than in males, representing 8.5% of all proteins detected (Fig. 6B, Additional file 2: Table 1). In males, 218 proteins were expressed at significantly higher levels as compared to female (3.6% of all proteins detected, Additional file 2: Table 2). Unbiased principal component analysis (PCA) of the 730 significantly affected proteins from the two-way ANOVA analysis revealed that the protein expression differences cluster by sex (Fig. 6C; Additional file 2: Tables 1–10); 164 proteins showed no sex difference in expression (Additional file 2: Table 3). Of these, 595 proteins showed a fold difference of 1.2 or greater (Fig. 6D) with more than 75% being detected at higher levels in females (Fig. 6E, Additional file 2: Table 4). Gene ontology (GO) enrichment analysis of the significantly affected proteins using the bioinformatic database DAVID [41, 42] was performed for GO-Molecular Function (MF), GO-Biological Processes (BP), and GO-KEGG pathways (Fig. 6F–H; Additional file 2: Tables 5–10). Hydrolase and catalytic activity, lipid metabolism, and metabolic pathways were identified within the top ten significant GO terms in molecular function, biological processes, and KEGG pathways, respectively, in females; male terms did not include lipid targeting outcomes.

Individual components of the ECS were assessed in the data set as the GO-BP analysis revealed that regulation of the endocannabinoid system was significantly higher in the female PAG versus male (*p* = 0.05) with a fold enrichment of 38.1. DAGLα, DAGLβ, MAGL, ABHD6, ABHD12, NAPE-PLD 7 isoforms, and CNR1 were detected; neither FAAH nor CNR2 was detected (Table 1). Female PAG contained significantly more MAGL and ABHD6 than males; these were the only differences reaching statistical significance in the ECS data set and showed a 38-fold enrichment as compared to the background gene set in DAVID GO analyses (Additional file 2: Tables 1–10). These data, together with the analytical chemistry, suggest that females have a dominance of ECS metabolism in the PAG as compared to males.

**Table 1:** Sex differences in PAG ECS proteome

	Accession	Max fold change over other sex	Anova (*p*)
*Detected greater in female*	ABHD6_MOUSE*	***1.51***	***0.03***
	MGLL_MOUSE*	***1.44***	***0.01***
	*NAPEP_MOUSE*	*1.34*	*0.93*
	PLD3A_MOUSE	1.11	0.46
	PLD2_MOUSE	1.18	0.56
	DGLB_MOUSE	1.05	0.56
	*DGLA_RAT;DGLA_MOUSE*	*1.01*	*0.85*
Detected greater in male	PHLD_MOUSE	1.52	0.15
	PLD1_RAT;PLD1_MOUSE	1.18	0.30
	PLD3_MOUSE	1.07	0.40
	PLD6_MOUSE	1.15	0.84
	*CNR1_RAT*	*2.49*	*0.13*
	CNRP1_MOUSE;CNRP1_RAT	1.02	0.82
	ABD12_MOUSE	1.02	0.79

Summary of endocannabinoid system (ECS) enzymes detected in greater quantity in either female or male PAG samples, as measured by quantitative proteomics. The 2-AG hydrolyzing enzymes MAGL and ABHD6 (bold) were detected at significantly higher fold-detection for females vs males (*ABHD6 female vs. male *p* = 0.03, MAGL female vs. male *p* = 0.01). Italics indicate study via IHC. CNR2 and FAAH were below the level of detection in this study.

Protein gene abbreviations: *ABHD6* alpha/beta-hydrolase domain containing 6; *MGLL* monoacylglycerol lipase; *NAPEP* *N*-acyl-phosphatidylethanolamine-specific phospholipase D; *PLD3A* phospholipase D3A; *PLD2* phospholipase D2; *DGLB* diacylglycerol lipase beta; *DGLA* diacylglycerol lipase alpha; *PHLD* phosphatidylinositol-glycan-specific phospholipase D; *PLD1* phospholipase D1; *PLD3* phospholipase D3; *PLD6* phospholipase D6; *CNR1* cannabinoid receptor 1; *CNRP1* cannabinoid receptor interacting protein 1; *ABD12* alpha/beta-hydrolase domain containing 12

## Discussion

The goal of this study was to define the organization of the ECS in neural regions associated with pain in the context of sex. Levels of the primary eCBs, AEA and 2-AG, differed between cortical, brainstem (i.e., lPAG), and trigeminal components (Vc and TG) in a sex-dependent manner. Despite increased levels of 2-AG detected within female trigeminal regions, 2-AG concentration was significantly decreased within female PAG. Furthermore, 2-AG concentration was not observed to be estrous stage dependent within the PAG. Unbiased, quantitative proteomic analysis confirmed sex differences in the PAG proteome such that detection of MAGL and ABHD6, the 2-AG hydrolyzing enzymes, was statistically higher in female than in male samples. Immunohistochemical analysis confirmed the female prevalence of NAPE-PLD, DAGL, and MAGL in the PAG. Moreover, the distributions of CB_1_R and CB_2_R were significantly different between the sexes as well, with CB_1_R lower in males than females in the V1M cortex and PAG. Together, these results suggest differences between the physiology of the male and female ECS in brain regions associated with migraine aura and descending pain modulation.

### AEA

Dysregulation of AEA signaling has been implicated in migraine, including chronic migraine and medication overuse headache (MOH) [43, 44], though this has been contested [45]. Peripheral actions of AEA at CB_1_R receptors inhibit trigeminal afferent release of neurotransmitters to mitigate headache pain and regulate vascular tone [46]. Levels of AEA are reduced in the plasma of chronic migraine and MOH patients [43]. In addition, female, but not male, migraineurs were reported to have increased activity of AEA transporters and FAAH in platelets, suggesting sex differences [47]. Measurement of AEA in the cerebrospinal fluid revealed that chronic migraineurs and patients with MOH had significantly lower levels as compared to controls or episodic migraineurs [48], suggesting widespread dysfunction of the AEA system in the CNS.

Notably, preclinical studies of AEA and its modulation have yielded promising results as a strategy to mitigate migraine. In male rats, following administration of nitroglycerin, an NO-donor used to induce headache pain [49, 50], AEA administration blocked changes in expression of TRPV1, NFKb, CRP, and NO [51] and reduced trigeminal activation [52]. Further, FAAH inhibitors were effective as both preventative and reversal agents against NO-induced headache [51, 53, 54]. Of importance, though NO-induced headache has a reported sexual dimorphism [55], the studies using this model were performed in males [51–54]. Current findings suggest that NAPE-PLD and FAAH are distributed differently among neuron, astrocyte, and glial populations as well. These observations, combined with clinical reports, indicate that sexually dimorphic responses to restoring physiological AEA tone with FAAH or AMT inhibitors warrant additional investigation with attention to female subjects [56].

Our data support sex differences in the levels of AEA in the V1M cortex, PAG, and TG, such that AEA was detected in greater quantities in female vs male samples in these regions. This may explain why Cupini et al. observed increases in AEA transport only within female migraineurs rather than males [47]. Furthermore, the increased concentration of AEA within female cortex corresponds with a significant decrease in female cortical FAAH-ir observed within the immunohistochemistry studies above. No significant sex differences were observed in NAPE-PLD-ir or FAAH-ir within the PAG, nor were sex differences in these enzymes detected within the proteomic analysis, therefore factors governing AEA concentration in male versus female PAG may require further examination.

### 2-AG

The role of 2-AG has been understudied in migraine and pain states. Clinical studies have shown decreased levels of 2-AG in the platelets of chronic migraineurs and MOH patients [57]; however, CSF levels of 2-AG were below the limit of detection in other studies [47, 48]. 2-AG acts on both cannabinoid receptors, leading to reduction in neurotransmitter release at glutamatergic and GABA-ergic synapses [11–15]. Thus, alterations in levels of 2-AG are pertinent to pain sensation in the V1M cortex and descending pain modulation in the PAG.

Our results show that 2-AG levels are significantly lower in the female PAG as compared to male PAG. This aligns with clinical data demonstrating decreases in levels of 2-AG within migraineurs [57], as well as increased migraine prevalence in the female population. However, these investigations were focused on CSF and platelets, which may not reflect important differences within discrete brain circuits relevant to pain transmission (i.e., PAG). Given the differences in PAG 2-AG levels between male and female rats, and clinical inconsistencies in reporting of 2-AG levels, it is possible that sex differences in the expression or activity of synthetic (DAGL) or degradative enzymes (MAGL, ABHD6) for 2-AG underlie the observed sex difference in 2-AG levels.

### DAGL

Preclinically, DAGLα expression is reported in the perisynaptic region of the dendritic spines of glutamatergic synapses [58], although cytosolic and nuclear DAGLα has also been described in cortical neurons [59]. Studies above observed DAGLα in neuronal soma and fibers, astrocytes, and microglia in both the V1M cortex and PAG. Recently, mutations in DAGLA, the gene for DAGLα, within the CNS were identified and implicated in neurological disorders in humans [60]. Furthermore, there is evidence that depletion of 2-AG via inhibition of DAGLα may be sufficient to trigger migraine-like pain in animals [61]. Thus, pathological reductions in the functional expression of DAGLα in females during pain states, including headache, may have a pronounced impact via decreased 2-AG signaling.

Our studies revealed that DAGLα expression was higher in female rat PAG as compared to male rat PAG using immunofluorescence. However, this increase was not detected within the unbiased proteomic study. Therefore, DAGL may not represent the driving factor in sex differences in 2-AG levels within the PAG, rather, the hydrolyzing enzymes (MAGL and ABHD6) may be involved.

### MAGL and ABHD6

In contrast to increases in DAGL synthesis of 2-AG, decreased levels of 2-AG in female PAG may reflect increased expression of the functional degradative enzymes MAGL and ABHD6. Both MAGL and ABHD6 degrade many monoacylglycerol species, including 2-AG [7, 8, 62–66]. MAGL and ABHD6 inhibitors have shown promise as pain therapeutics [61, 66–71] and in treating neurological disorders [7, 8, 66], respectively. One study investigated the contribution of MAGL to NO-induced headache, but results were not obtained in both sexes [54]. Mechanistically, the effects of the inhibitors have correlated to increased 2-AG levels and CBR signaling while attenuating eicosanoid signaling [12, 72].

Present proteomic and immunohistochemical data indicate that 2-AG degradation may be higher in females as compared to males in the PAG, thus generating the observed decreased in female 2-AG levels within the PAG. Notably, ABHD6 has been shown to have DAGL activity in addition to the breakdown of monoacylglycerols [73], and this may contribute to sex differences in 2-AG levels within PAG. Our findings suggest that sex differences in the prevalence of disorders where the PAG is implicated, including migraine and other pain states [74–82], may result from inherent differences in degradation of 2-AG. Sex differences in signaling of 2-AG at the eCB receptors within pain modulatory pathways may then influence the frequency and intensity of pain signals sent to the somatosensory cortex.

### CB receptors

In the context of pain, preclinical data support sex differences in responsivity to cannabinoid receptor agonists including ∆^9^-tetrahydrocannabinol (THC), cannabidiol, beta-caryophyllene (BCP), WIN55-212-2, and CP55-940 [82–85]. In support of the theory of CED, Kandasamy et al. demonstrated THC, a CB_1/2_ agonist, ameliorated the effects of induced migraine in rats, and that this effect was attenuated on administration of a CB_1_ antagonist [83]. CB_2_R expression in the CNS has been controversial, and current clinical literature supports a role for it during inflammation [39, 86–90].

Agonism of the CB receptors is being pursued for several neurological disorders, including migraine [6, 90–95]. In addition, more than 300 publications report sex differences in pathologies where the ECS plays a regulatory role. Our data now reveal that these sex differences are discernable in discrete brain regions and cell types within pain-relevant regions of the CNS. Thus, cannabinoid pharmacology may have anatomical and circuit-based differences between the sexes in basal and pathological states.

Female cortical and lPAG sections showed significantly higher CB_1_R immunoreactivity as compared to the respective male tissue. Male CB_2_R expression was largely in neurons, whereas female colocalization was primarily in glial cells. These data suggest that differences in the literature reporting CNS expression of CB_2_R would benefit from analysis by sex and that CB_2_R in the CNS may play different roles between male and female subjects. Given the interest in developing CB_2_R targeting therapeutics for pain and neurological disorders, these sex differences in the biological role of CB_2_R are important [96, 97].

### Estrous stage dependence of the ECS

Clinically, menstrual migraine has been associated with declining levels of estrogen [98]. While the rat estrous cycle is not a perfect representation of the human female menstrual cycle, our data offer insights into the role that eCB signaling may play in these hormonal pain states. In accordance with previously published work [26, 27], female tissue harvested according to the estrous cycle stages (proestrus, estrus, metestrus, diestrus) displayed variations in levels of 2-AG and AEA. The most profound change was observed within V1M cortex, wherein AEA levels dramatically decreased from proestrus to estrus (roughly 60–5 pmol/g). Previous work has shown that estrogen levels peak within the rat proestrus stage, followed by a rapid drop-off as the rats move into estrus [99]. This decline in estrogen levels correlates with the observed decrease in cortical AEA levels, therefore decreased AEA signaling may play a role in menstrual migraine development. However, as the decrease in AEA concentration was not observed in alternate CNS regions or within metestrus and diestrus, this difference warrants additional investigation. The other estrous stage dependence that was detected via the primary ANOVA analysis occurred within the Vc. 2-AG levels within Vc increased according to the progressive estrous cycle stages, peaking within diestrus. As diestrus has been associated with increased trigeminal nociception in rats [100], it is possible that 2-AG levels rise to compensate for increased noxious signaling during this stage. Notably, this is the only region in which 2-AG concentration varied according to estrous cycle stage. Given these variations, estrous staging of female rats remains pertinent to investigations of the ECS.

### Further implications of ECS sex differences

While the above studies focused on pain-relevant regions within the CNS, the observed differences in levels of 2-AG and AEA, as well as proteins in the ECS, may apply to other reported sex differences in eCB-regulated biology. Reports have shown greater cannabis use in males, despite females demonstrating greater risk of developing cannabinoid use disorder [101]. Dronabinol, a cannabinoid agonist, was shown to delay gastric emptying to a greater extent in females as compared to males [102]. Together, these reports may indicate that females display an increased susceptibility to the effects of cannabinoid agonists; however, in a study of cannabinoid-mediated food intake, male rats displayed greater increases in energy intake than female rats following cannabinoid agonism [103]. As was the case in this study, it is likely that these differing effects result from regional sex differences in eCB biology. We observed lower levels of 2-AG in the PAG, which correlates with increased pain susceptibility in the female population. Similarly, regional sex differences in the ECS of the limbic system, the enteric nervous system, and the hypothalamic system may underlie sex differences in cannabinoid use disorder, gastric motility, and feeding behaviors, respectively [101–103].

### ECS distribution in neurons, glia, and immune cells

Throughout the immunohistochemical analyses, it was observed that for all proteins in the ECS, colocalization with Nissl (neuronal soma), GFAP (astrocyte marker), and Iba1 (microglial marker) was limited to 40–70%. Thus, 30–60% of the ECS proteins were observed independent of these markers. It is possible that a portion of the observed proteins that were non-localized were due to aberrant pixel detection, either from background noise on the image or from image artifacts. However, there is evidence within the literature for the role of these proteins at synaptic terminals, which may have eluded the selected cell markers [6–8, 42]. Proteins present in the neurovascular and extracellular spaces likely would not be colocalized with our cellular markers. For all immunohistochemical analyses, the overall staining/immunoreactivity with these biomarkers of cellular identity (neuron, astrocyte, or microglia) were the same between regions and each sex, confirming that regional comparisons were appropriately controlled for cell types and number (Additional file 1: Fig. 1).

## Limitations

Though carefully designed, this study does have some limitations. First, immunohistochemistry with antibodies targeting proteins of interest was used. Antibodies, particularly those against the cannabinoid receptors, are reported to be non-specific and unreliable [104, 105]. To combat this, we paired imaging with an unbiased quantitative proteomics approach. This unbiased method allowed for determining presence of proteins in each region by sex, but itself is limited in spatial resolution by cell type. This lack of detection may reflect masking by proteins with higher abundance (i.e., low signal-to-noise). Second, all tissue was obtained from Sprague–Dawley rats between 7 and 8 weeks old. As the ECS is implicated in neural development [27, 106], it is possible that sex differences identified here become more/less prominent during aging. Future studies should incorporate newly developed fluorescent chemical probes for labeling these CBRs to confirm the present observations [107, 108] and investigate the sex difference in the ECS with aging females. Finally, the immunohistochemical and proteomic analyses were performed on female rats without regard to estrous stage. While this does present a limitation to these data, 2-AG levels did not display estrous stage dependence within V1M cortex or PAG, therefore it is the belief of the authors that the above conclusions with reference to the 2-AG system are sound. AEA levels did display estrous stage dependence within V1M cortex and PAG, therefore repeat studies of NAPE-PLD-ir and FAAH-ir with females in particular estrous stages are likely warranted.

## Perspectives and significance

Neuropathological disorders such as chronic pain, migraine, and multiple sclerosis are associated with a large sex difference in prevalence. Moreover, the pharmacology of analgesics, including cannabinoids, is documented as having sex-selective outcomes. Our observations suggest that sex differences exist with regional and cellular selectivity in the neuroanatomical arrangement of the ECS within the V1M cortex, PAG, and trigeminal regions. The V1M cortex, a locus of CSD induction, showed the greatest amount of AEA in females. If AEA signaling at cortical CB_1_R and CB_2_R is primarily inhibitory, this may offer compensatory protection against pain sensation and migraine-associated cortical spreading depression in females. Levels of 2-AG were significantly lower in female PAG as compared to male PAG. As the PAG participates in descending pain modulation, and 2-AG has been shown to decrease presynaptic signaling, decreases in 2-AG levels may predispose the female system to increased nociception. This sexual dimorphism of the ECS within pain modulatory pathways may underlie the sex differences observed in pain etiologies and cannabis pharmacology and indicates that both pain and pharmacological investigations should take sex into account when studying the ECS.

## Supplementary Information

**Additional file 1: Figure 1.** Regional analysis. **A** Serial sections from rats’ brains were obtained at a thickness of 30um, moving posteriorly from approximately Bregma -7mm with PAG imaging in the lateral PAG. **B** Quantification of the relative area immunoreactive in each field for Nissl (neuronal soma), GFAP (astrocytes), and Iba1 (microglia) was statistically similar between regions and sex.

**Additional file 2: Table 1.** Proteins more prevalent in female PAG. A total of 3396 proteins were more prevalent in female PAG. Of the 3396 proteins with female prevalence, 512 were present at statistically significantly higher levels than in males (highlighted in grey), representing 8.5% of all proteins detected. **Table 2.** Proteins more prevalent in male PAG. A total of 2449 proteins were more prevalent in male PAG. In males, 218 proteins were expressed at significantly higher levels as compared to female (3.6% of all proteins detected) (highlighted in grey). **Table 3.** List of proteins detected in PAG samples without sex-differences. 164 proteins showed no sex difference in expression. **Table 4.** Fold differences. 595 proteins showed a fold difference of 1.2 or greater with more than 75% being detected at higher levels in female PAG. **Table 5.** GO-MF Female analysis. Gene ontology (GO) enrichment analysis of the significantly affected proteins in female PAG using the bioinformatic database DAVID was performed for GO-Molecular Function (MF). The top 10 category is highlighted. **Table 6.** GO-BP female analysis. Gene ontology (GO) enrichment analysis of the significantly affected proteins in female PAG using the bioinformatic database DAVID was performed for GO-Biological Processes (BP). The top 10 category is highlighted. **Table 7.** GO-KEGG female analysis. Gene ontology (GO) enrichment analysis of the significantly affected proteins in female PAG using the bioinformatic database DAVID was performed for GO-KEGG pathways. The top 10 category is highlighted. **Table 8.** GO-MF male analysis. Gene ontology (GO) enrichment analysis of the significantly affected proteins in male PAG using the bioinformatic database DAVID was performed for GO-Molecular Function (MF). The top 10 category is highlighted. **Table 9.** GO-BP male analysis. Gene ontology (GO) enrichment analysis of the significantly affected proteins in male PAG using the bioinformatic database DAVID was performed for GO-Biological Processes (BP). The top 10 category is highlighted. **Table 10.** GO-KEGG male analysis. Gene ontology (GO) enrichment analysis of the significantly affected proteins in male PAG using the bioinformatic database DAVID was performed for GO-KEGG pathways. The top 10 category is highlighted.

## Data Availability

The datasets used and/or analyzed during the current study are available from the corresponding author on reasonable request.
