# Supplementary figures and images for: Sex differences in the expression of the endocannabinoid system within V1M cortex and PAG of Sprague Dawley rats
Source: Biol Sex Differ. 2021 Nov 8;12:60. doi: 10.1186/s13293-021-00402-2 (PMC8577021; doi:10.1186/s13293-021-00402-2)

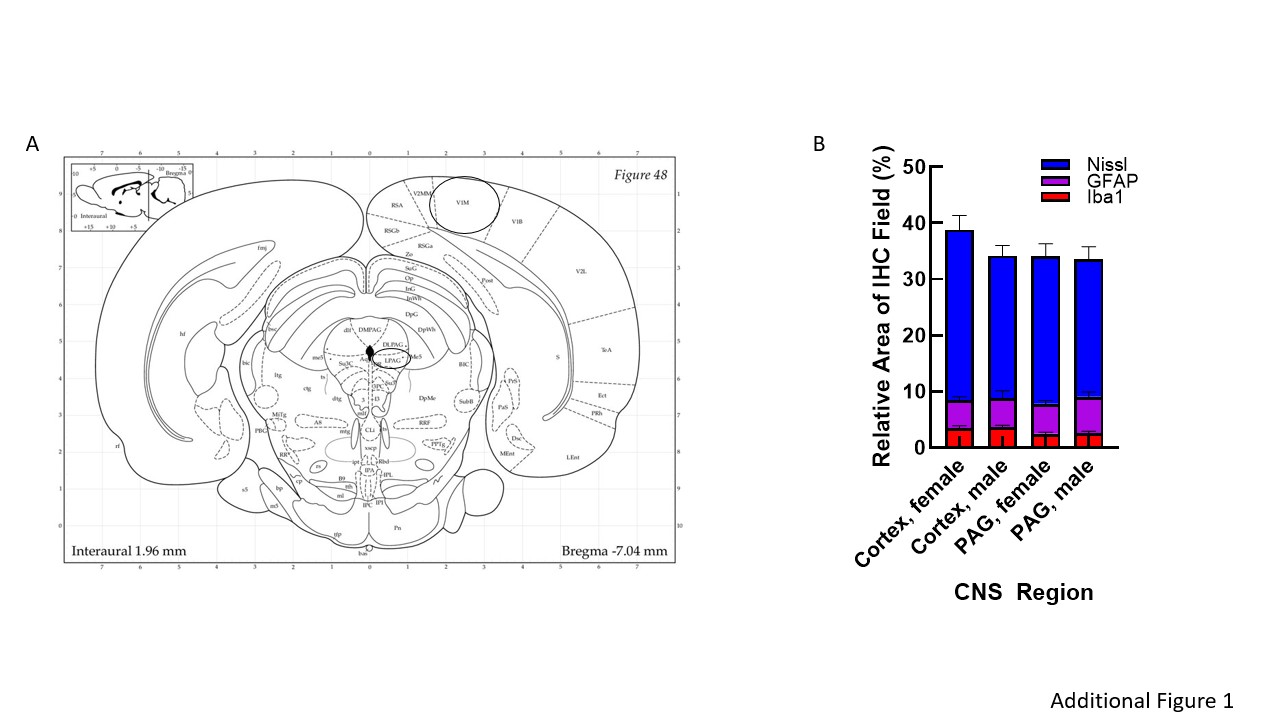

Supplement: Supplementary file 1 — Additional file 1: Figure 1. Regional analysis. A Serial sections from rats’ brains were obtained at a thickness of 30um, moving posteriorly from approximately Bregma -7mm with PAG imaging in the lateral PAG. B Quantification of the relative area immunoreactive in each field for Nissl (neuronal soma), GFAP (astrocytes), and Iba1 (microglia) was statistically similar between regions and sex. [file 13293_2021_402_MOESM1_ESM.jpg]
